# Analysis of Effects of Meteorological Factors on Dengue Incidence in Sri Lanka Using Time Series Data

**DOI:** 10.1371/journal.pone.0063717

**Published:** 2013-05-09

**Authors:** Kensuke Goto, Balachandran Kumarendran, Sachith Mettananda, Deepa Gunasekara, Yoshito Fujii, Satoshi Kaneko

**Affiliations:** 1 Department of Eco-epidemiology, Institute of Tropical Medicine, Nagasaki University, Nagasaki City, Nagasaki Prefecture, Japan; 2 Department of Public Health, Faculty of Medicine, University of Kelaniya, Gampaha District, Western Province, Sri Lanka; 3 Department of Paediatrics, Faculty of Medicine, University of Kelaniya, Gampaha District, Western Province, Sri Lanka; 4 Department of Biochemistry and Clinical Medicine, Faculty of Medicine, University of Kelaniya, Gampaha District, Western Province, Sri Lanka; Kenya Medical Research Institute-Wellcome Trust Research Programme, Kenya

## Abstract

In tropical and subtropical regions of eastern and South-eastern Asia, dengue fever (DF) and dengue hemorrhagic fever (DHF) outbreaks occur frequently. Previous studies indicate an association between meteorological variables and dengue incidence using time series analyses. The impacts of meteorological changes can affect dengue outbreak. However, difficulties in collecting detailed time series data in developing countries have led to common use of monthly data in most previous studies. In addition, time series analyses are often limited to one area because of the difficulty in collecting meteorological and dengue incidence data in multiple areas. To gain better understanding, we examined the effects of meteorological factors on dengue incidence in three geographically distinct areas (Ratnapura, Colombo, and Anuradhapura) of Sri Lanka by time series analysis of weekly data. The weekly average maximum temperature and total rainfall and the total number of dengue cases from 2005 to 2011 (7 years) were used as time series data in this study. Subsequently, time series analyses were performed on the basis of ordinary least squares regression analysis followed by the vector autoregressive model (VAR). In conclusion, weekly average maximum temperatures and the weekly total rainfall did not significantly affect dengue incidence in three geographically different areas of Sri Lanka. However, the weekly total rainfall slightly influenced dengue incidence in the cities of Colombo and Anuradhapura.

## Introduction

Dengue fever (DF) and dengue hemorrhagic fever (DHF) outbreaks occur in most tropical and subtropical regions and are the most important emerging arboviral diseases worldwide. The endemic area for dengue extends over 60 countries [1]–[3]. It is estimated that tens of millions of people develop DF, and approximately 500,000 people develop DHF. In addition, dengue causes more than 20,000 deaths per year, and approximately 2.5 billion people live in dengue-endemic countries [4]. Dengue virus infection in humans causes a spectrum of illness, ranging from asymptomatic or mild febrile illness to severe and fatal hemorrhagic disease [5]. The most severe cases are caused by a flavivirus with four distinct serotypes: DV-1, DV-2, DV-3, and DV-4 [6], [7]. The spectrum of clinical illness includes undifferentiated fever, classic DF, DHF, and dengue shock syndrome (DSS).

In Sri Lanka, although dengue is endemic, the case fatality ratio (CFR) is below 1%; the number of adult cases have increased recently [8]. Twenty-five notifiable diseases, including cholera, plague, yellow fever, and dengue, are reported by Medical Officers of Health in Sri Lanka [9]. Dengue cases are reported from all over Sri Lanka; however, the western part of the country is most affected. Dengue was serologically confirmed in Sri Lanka in 1962, the first outbreak was reported in 1965 [10], and dengue epidemics in Sri Lanka have occurred almost every other year since 2002 [8].

At present, the causes and influencing factors of dengue epidemics are unknown in Sri Lanka. Previous studies demonstrate statistically significant associations between infectious diseases and meteorological variations such as rainfall and temperature. The effects of climate change on the endemics of infectious diseases such as cholera, malaria, and plague have been recognized [11]–[20].

Time series analyses are often used in studies of the relationship between meteorological factors and disease and are most successful when data have been accumulated over long periods. However, it is extremely difficult to collect such meteorological and health data in developing countries. Although daily outcome data are desirable for time series analysis, obtaining such data from most developing countries is impossible [21]. Hence, most time series analyses use monthly or annual data.

Fortunately, in Sri Lanka, the number of dengue cases is reported from all over the country, and meteorological data are collected and made readily available. Importantly, both these databanks contain weekly data. Thus, in the present study, we examined the effects of meteorological factors on dengue outbreak in Sri Lanka using time series analysis. Studies of dengue in Sri Lanka are few [22]–[26], and none of these have considered the effects of meteorological factors on dengue in Sri Lanka using time series analysis.

We aimed to quantify in detail the association between meteorological variables and the frequency of notified cases of dengue in three geographically distinct areas (Ratnapura, Colombo, and Anuradhapura) of Sri Lanka using time series analyses of weekly data.

## Materials and Methods

### Study Area

**Figure 1:**
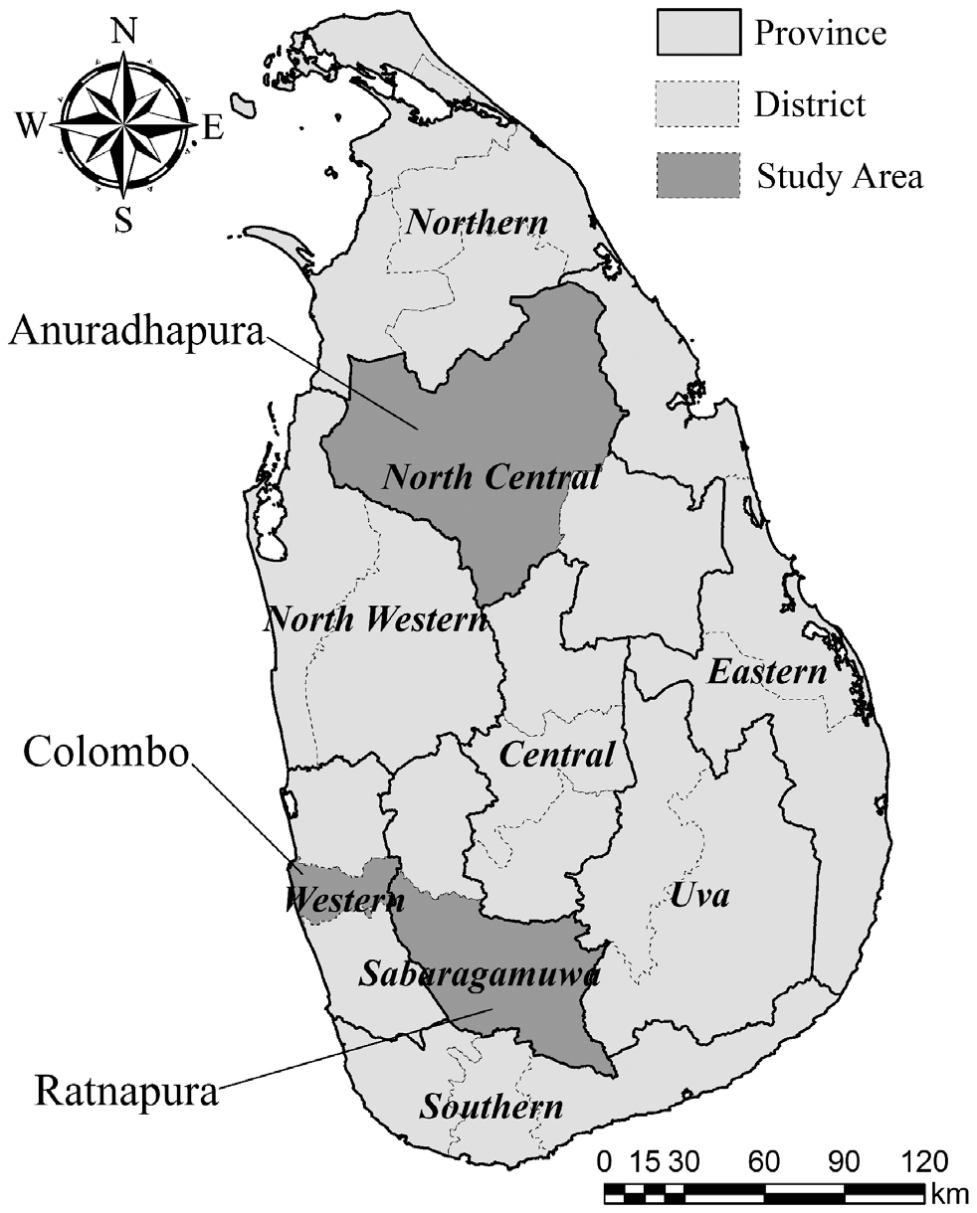
Study Locations.

The climate of Sri Lanka is characterized as tropical and is traditionally divided into three climatic zones. In a large number of previous studies, time series analyses were performed in one study area owing to difficulties in data collection. However, it is desirable to compare several study areas when investigating the effect of meteorological factors on infectious diseases. In this study, using existing surveillance data, we quantified the association between meteorological variables and dengue incidence in three climatically different areas, namely Ratnapura, Colombo, and Anuradhapura districts (Figure 1).

Ratnapura district is located in the South-western part of Sri Lanka and is 101 km from Colombo in the Sabaragamuwa Province, which has a tropical rainforest climate and a population of 1 million. The average annual precipitation is approximately 4,000–5,000 mm in the valley (21 m above sea level) of the River Kalu Ganga, and the average temperature varies from 24°C to 35°C. Colombo district is the largest in Sri Lanka and is the administrative capital of the province located in the country's west coast. This region has a tropical monsoon climate and a population of 2.3 million. The average annual precipitation is approximately 2,400 mm, and the average temperature varies 28°C. Anuradhapura district is the capital of the North Central Province and one of the ancient capitals of Sri Lanka. It is located 206 km from Colombo. This district has a hot tropical climate and a population of 0.7 million. The average annual precipitation is approximately 1,300 mm, and the average temperature varies from 20°C to 30°C.

### Data Collection

This study covers dengue incidence and meteorological data from 2005 to 2011 (7 years). However, complete data were only collected until the 39^th^ week, 52^nd^ week, and 48^th^ week of 2011 from Ratnapura, Colombo, and Anuradhapura, respectively. Meteorological data were collected by the Department of Meteorology in Sri Lanka and included the daily maximum temperature and total rainfall, which were acquired from Ratnapura (6.68N, 80.40E, 34.4 m), Colombo (6.90N, 79.87E, 7.3 m), and Anuradhapura (8.35N, 80.38E, 92.5 m) weather stations managed by the Department of Meteorology. We obtained these archived data from them. Because time series analyses were performed using weekly disease data, daily meteorological data were converted to weekly data.

Dengue incidence is reported in Sri Lanka through a national network that covers the whole country. These values are published as weekly epidemiological reports (WERs) by the Epidemiology Unit, Ministry of Health, Sri Lanka. In this study, weekly dengue incidence data were obtained from clinically diagnosed cases at this unit and excluded laboratory surveillance. In addition, data included both DF and DHF and were not divided into the four viral serotypes DV-1, DV-2, DV-3, and DV-4.

### Data Analysis

The ordinary least squares (OLS) method and the vector autoregressive model (VAR) were used in this study to examine the association between meteorological variables and the incidence of dengue from 2005 to 2011. In this study, OLS regression analyses were performed, and if serial correlations were revealed, these analyses were followed by VAR. VAR is one of the most flexible models for analyses of multivariate time series. The main advantage of VAR is that multivariate variables are both explained and explanatory variables. Hence, this model performs more accurate predictions using the relations between multiple variables [27]. This model is extremely popular in economics and elucidates underlying causal mechanisms using the Granger causality test [28], [29]. This test determines whether past variables can provide predictive information.

In addition, we also used impulse response function (IRF) to identify shock reactions to the maximum temperature and total rainfall. IRF tracks the impact of all variables on the others in the system [30], [31].

All analyses were performed using STATA version 12 (StataCorp. LP, College Station, USA).

## Results

### Descriptive Analysis

**Figure 2:**
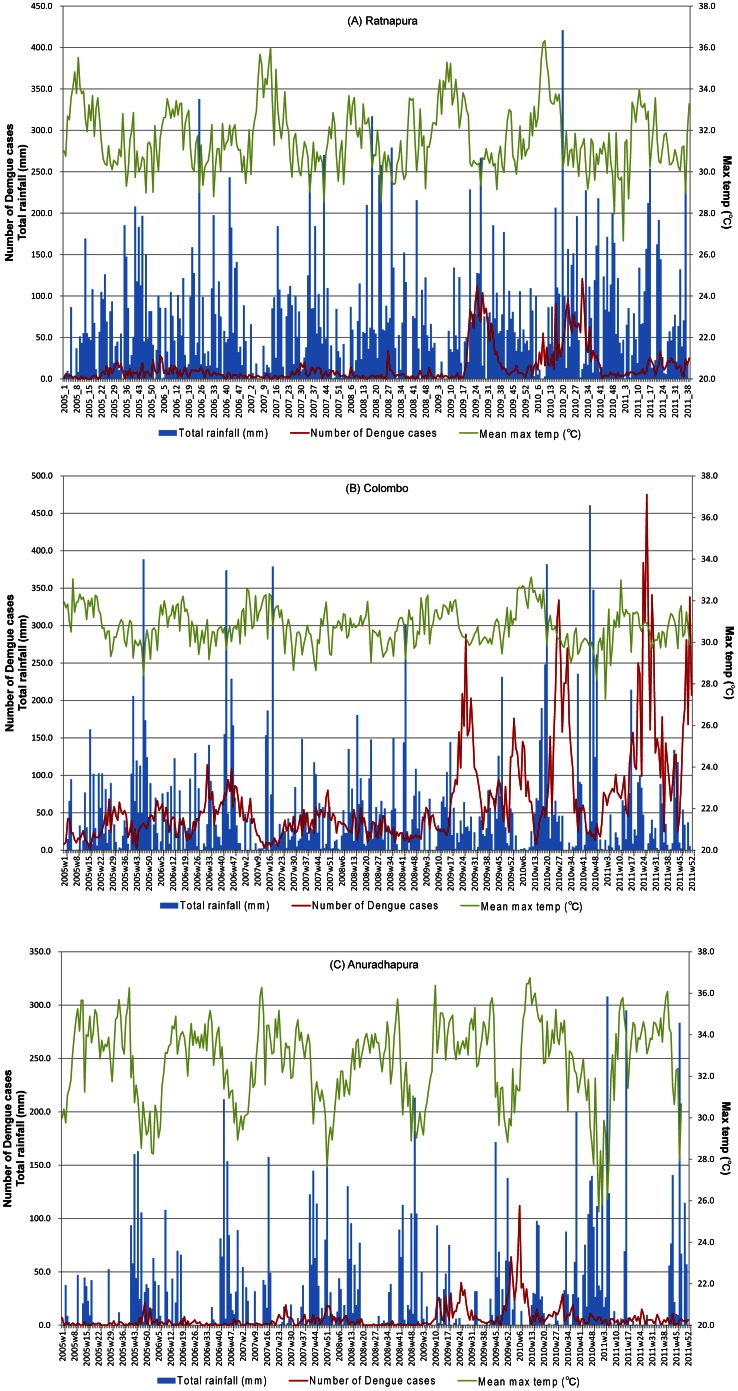
Meteorological variables and the number of dengue cases. (A) Ratnapura (B) Colombo (C) Anuradhapura.

Characteristics of meteorological variables and dengue incidence differed between study areas (Figure 2). Weekly average maximum temperatures and rainfall at Ratnapura, Colombo, and Anuradhapura were 31.6°C, 30.8°C, and 32.7°C and 71.1 mm, 48.0 mm, and 27.1 mm, respectively. As indicated in Figure 2, all areas had regular changes in weekly maximum temperatures, with small regularity in Ratnapura and large regularities in Anuradapura. Temperature differences throughout the year in Ratnapura and Anuradapura were more extreme than those in Colombo.

Total numbers of dengue cases recorded between 2005 and 2011 were 2720, 22231, and 2090 in Ratnapura, Colombo, and Anuradapura, respectively. Outbreaks occurred in 2009 and 2010 in all three areas, although in Colombo, the outbreak was most remarkable and continued in 2011.

### Linear Regression Analysis

OLS regression analyses were initially performed for each area and are shown in Table 1, 2, and 3. When the maximum temperature correlated with the total rainfall, OLS regression analysis could not be used. As is evident from the Durbin–Watson statistics from the three areas, maximum temperatures and total rainfall were serially correlated (Ratnapura, DW = 0.359; Colombo, DW = 0.446; Anuradhapura, DW = 0.566). Low Durbin–Watson statistics indicate positive serial correlations between variables.

**Table 1:** OLS regression analysis (Ratnapura).

Source	SS	df	MS	Number of observations = 352
Model	5056.87238	2	2528.43619	F (2, 349) = 6.04
Residual	146061.446	349	418.514171	Prob>F = 0.0026
Total	151118.318	351	460.536519	R-squared = 0.0335
				Adj R-squared = 0.0279
				Root MSE = 20.458

dengue	Coeff.	Std. Err.	t	P>| t |	95% Conf.	Interval
Temp	−1.735021	0.7685898	−2.26	0.025	−3.246672	−0.2233708
Rainfall	0.0261063	0.0179327	1.46	0.146	−0.0091634	0.061376
_cons	67.12854	24.89586	2.70	0.007	18.16375	116.0933

**Table 2:** OLS regression analysis (Colombo).

Source	SS	df	MS	Number of observations = 365
Model	69816.8803	2	34908.4401	F (2, 349) = 7.96
Residual	1588380.42	362	4387.79123	Prob>F = 0.0004
Total	1658197.30	364	4555.48710	R-squared = 0.0421
				Adj R-squared = 0.0368
				Root MSE = 66.24

dengue	Coeff.	Std. Err.	t	P>| t |	95% Conf.	Interval
Temp	−12.65323	4.052452	−3.12	0.002	−20.62253	−4.683925
Rainfall	−0.1823936	0.0544296	−3.35	0.001	−0.2894315	−0.0753558
_cons	464.0644	125.5453	3.70	0.000	217.1746	710.9541

**Table 3:** OLS regression analysis (Anuradhapura).

Source	SS	df	MS	Number of observations = 361
Model	391.735052	2	195.867526	F (2, 349) = 1.87
Residual	37561.8328	358	104.921321	Prob>F = 0.1561
Total	37953.5679	360	105.426577	R-squared = 0.0103
				Adj R-squared = 0.0048
				Root MSE = 10.243

dengue	Coeff.	Std. Err.	t	P>| t |	95% Conf.	Interval
Temp	−0.4547149	0.2908249	−1.56	0.119	−1.026655	0.117225
Rainfall	−0.0209214	0.0124478	−1.68	0.094	−0.0454015	0.0035586
_cons	21.32583	9.640052	2.21	0.028	2.308586	40.34308

Because positive serial correlations between the maximum temperature and total rainfall values were found in all areas using Durbin–Watson statistics, we performed OLS regression analyses using differences between variables without constant terms. Serial correlations were detected using the Breusch–Godfrey test (Ratnapura, Prob>chi2 = 0.000; Colombo, Prob>chi2 = 0.000; Anuradhapura, Prob>chi2 = 0.000) and Durbin's alternative test (Ratnapura, Prob>chi2 = 0.000; Colombo, Prob>chi2 = 0.000; Anuradhapura, Prob>chi2 = 0.000). Likewise, in OLS regression analysis using the lag model, both the Breusch–Godfrey test (Ratnapura, Prob>chi2 = 0.000; Colombo, Prob>chi2 = 0.000; Anuradhapura, Prob>chi2 = 0.001) AND Durbin's alternative test (Ratnapura, Prob>chi2 = 0.000; Colombo, Prob>chi2 = 0.000; Anuradhapura: Prob>chi2 = 0.001) identified serial correlations. Thus, OLS regression analyses were inappropriate for this study.

### Time Series Analysis

**Figure 3:**
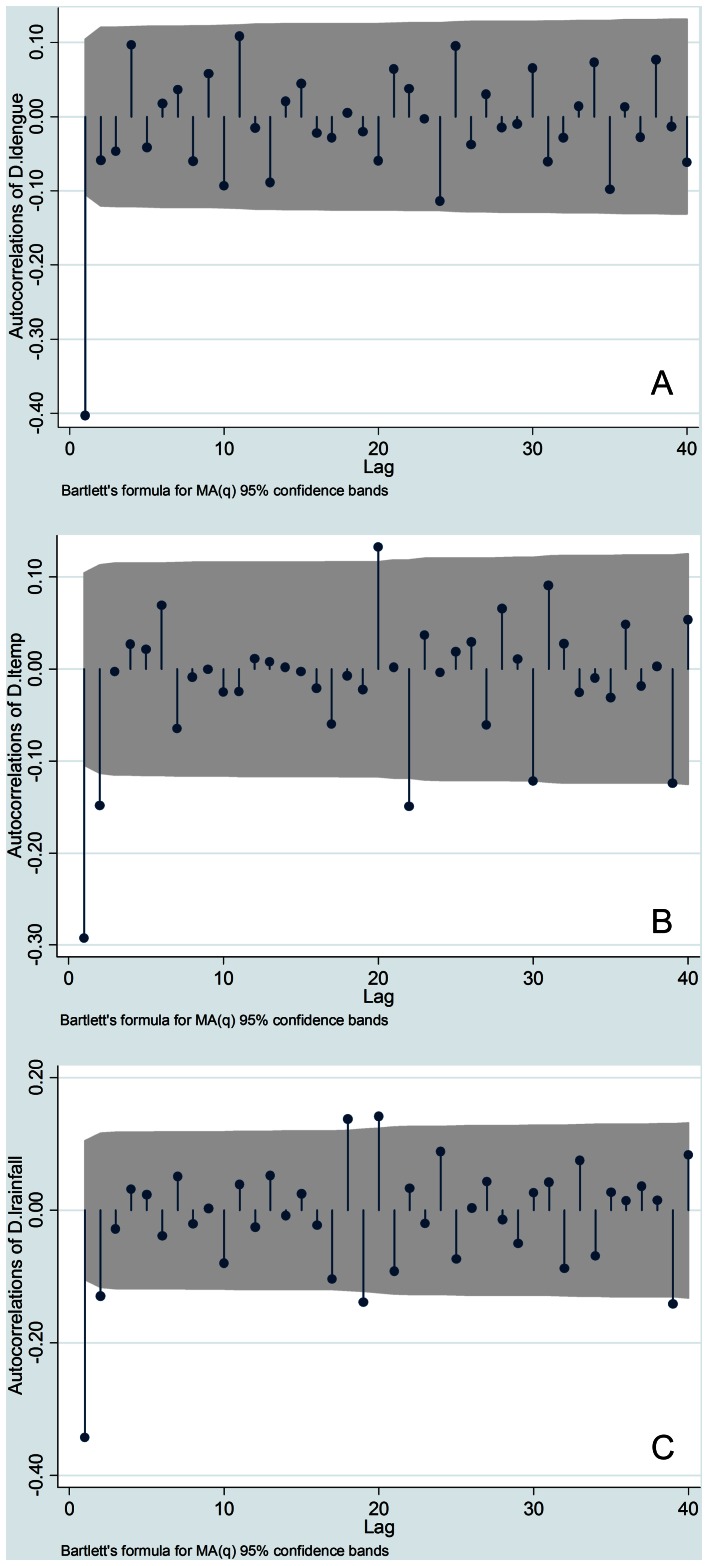
The correlogram of difference series data for all variables in Ratnapura. (A) Logarithm of dengue incidence (B) Logarithm of maximum temperature (C) Logarithm of total rainfall.

**Figure 4:**
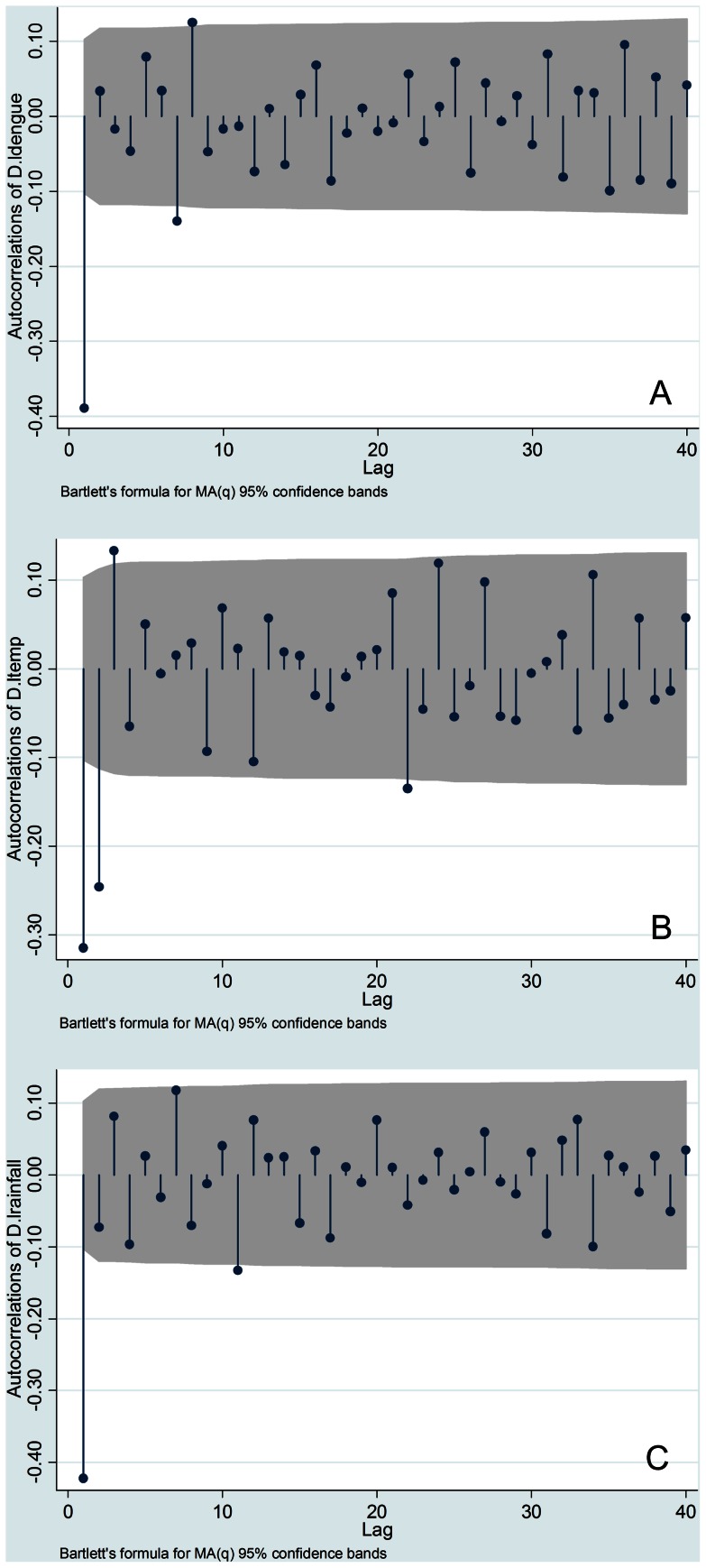
The correlogram of difference series data for all variables in Colombo. (A) Logarithm of dengue incidence (B) Logarithm of maximum temperature (C) Logarithm of total rainfall.

**Figure 5:**
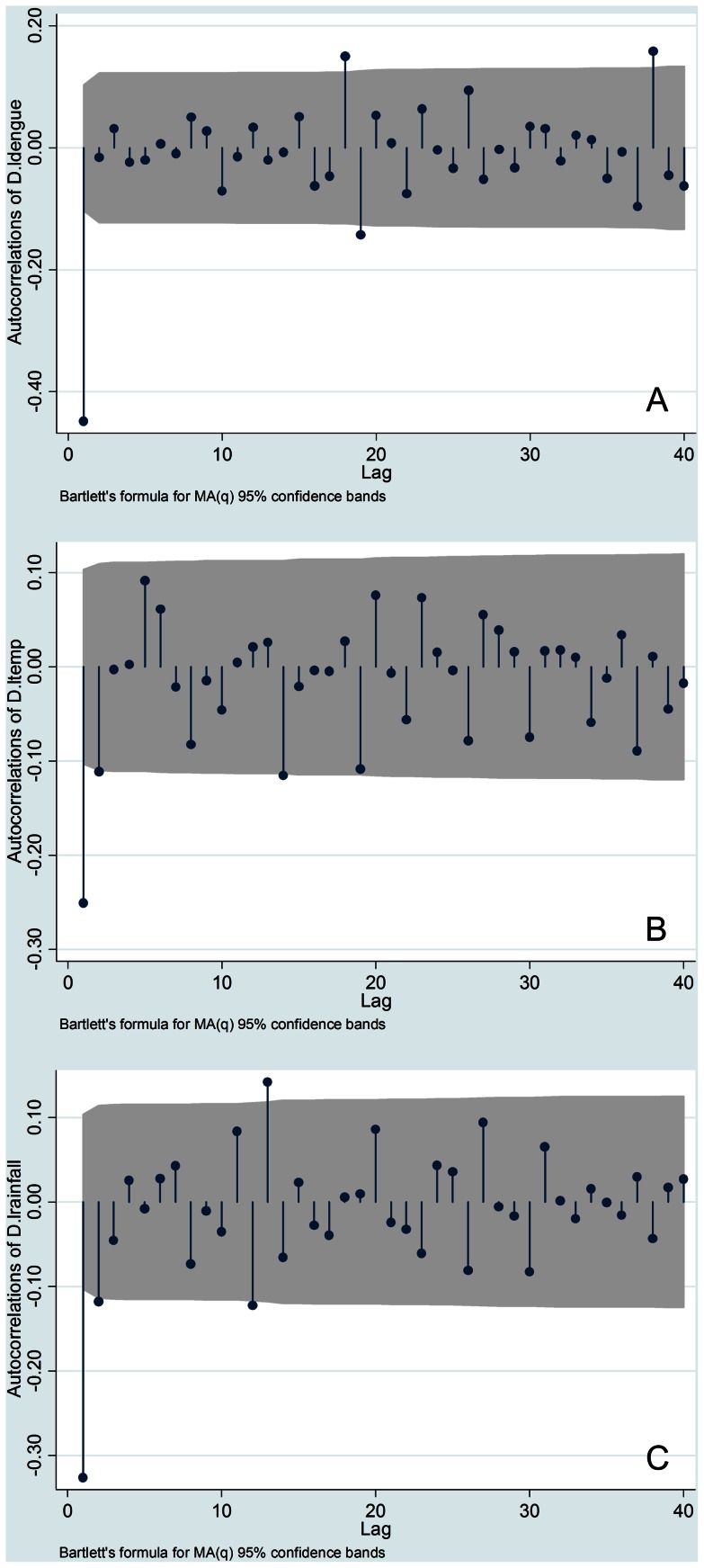
The correlogram of difference series data for all variables in Anuradhapura. (A) Logarithm of dengue incidence (B) Logarithm of maximum temperature (C) Logarithm of total rainfall.

To test the assumption that time series data represent a stationary process, a test for stationary processes was performed before time series analysis. The Dickey–Fuller GLS unit root test indicated that the original series of each variable were non-stationary processes in all three areas, with the exception of the total rainfall at Ratnapura and Colombo. In addition, as shown in Figures 3, 4, and 5, correlograms (autocorrelation at different lags) for all variables suggest that these were all first-difference stationary processes. Consequently, in this study, VAR was used to estimate first difference series data for all variables, excluding the total rainfall at Ratnapura and Colombo.

To determine the appropriate number of lags to be used in VAR, the final prediction error (FPE) and the Akaike Information Criterion (AIC) were used as common selection criteria. Both FPE and AIC selected a lag of four in Ratnapura (FPE = 0.0001179; AIC = 1077038), a lag of four in Colombo (FPE = 0.000315; AIC = 0.452156), and a lag of three in Anuradhapura (FPE = 0.004152; AIC = 3.02952).

As shown in Table 4, we performed Granger causality tests at the level of both variable and first differences. These tests showed that dengue incidence, the maximum temperature, and the total rainfall were independent of each other, although the total rainfall influenced dengue incidence in Colombo and Anuradhapura (Colombo, p = 0.051; Anuradhapura, p = 0.058).

**Table 4:** Granger causality test.

Equation	Excluded	Chi2			Prob>chi2		
		Ratnapura	Colombo	Anuradhapura	Ratnapura	Colombo	Anuradhapura
The Number of Dengue	Maximum Temperature	1.31980	0.61225	0.09922	0.251	0.434	0.753
The Number of Dengue	Total Rainfall	0.45196	3.79430	3.58700	0.501	0.051	0.058
The Number of Dengue	All	0.33810	3.79760	3.64450	0.512	0.150	0.162
Maximum Temperature	The Number of Dengue	0.10739	0.06836	2.85560	0.743	0.794	0.091
Maximum Temperature	Total Rainfall	0.35354	0.01394	0.38072	0.532	0.906	0.537
Maximum Temperature	All	0.47130	0.08630	3.03240	0.790	0.958	0.220
Total Rainfall	The Number of Dengue	0.14717	1.33500	0.30285	0.701	0.248	0.582
Total Rainfall	Maximum Temperature	0.04101	2.59390	2.53710	0.840	0.107	0.111
Total Rainfall	All	0.18020	3.64130	2.95590	0.914	0.162	0.228

Notes: Ratnapura and Colombo: Lags: 4. First difference series data of all variables excluding total rainfall. Anuradhapura: Lags: 3. First difference series data of all variables.

**Figure 6:**
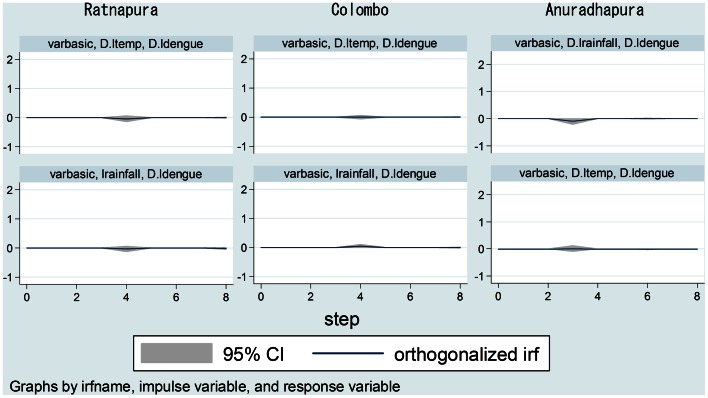
Impulse response functions.

IRF analyses presented in Figure 6 describe the influence of shock variables on the other endogenous variables in VAR. These analyses indicate that shocks of the maximum temperature and total rainfall had no effect on dengue incidence in any of the study areas.

## Discussion

This manuscript defines the influence of meteorological factors on dengue incidence using time series analysis of the weekly average maximum temperature and total rainfall from 2005 to 2011 in three geographically distinct areas of Sri Lanka: Ratnapura, Colombo, and Anuradhapura. In this study, we conducted time series analyses using OLS regression followed by VAR in each of the three areas. To the best of our knowledge, this is the first study to examine the impact of meteorological variables on dengue incidence in Sri Lanka using time series analyses based on VAR. In addition, such analyses of weekly data from three geographically distinct areas are extremely rare.

The analyses in this study led to the conclusion that the weekly average maximum temperature and total rainfall do not significantly affect dengue incidence in Ratnapura, Colombo, or Anuradhapura. However, the total weekly rainfall slightly influenced dengue incidence in Colombo and Anuradhapura (Colombo, p = 0.051; Anuradhapura, p = 0.058).

The results of this study differ from those of previous studies that indicate an association between meteorological variables and dengue incidence [32]–[35]. Most of these published studies suggest that temperature or rainfall contribute to the incidence of dengue, particularly increased rainfall. However, these results are dependent on the study area and country. In contrast, the present study indicates no such relationship between dengue incidence and rainfall. Indeed, data from Ratnapura, which has extremely high average annual precipitation (approximately 4,000–5,000 mm), gave a high p value (p = 0.701) compared with the other two areas. Likewise, the weekly average total rainfall calculated in descriptive analyses of this study was also the highest among the three areas (71.1 mm). VAR considered the impact of the total rainfall on dengue incidence, including gradual changes in the total rainfall. These data indicate that high rainfall or increased total rainfall does not always elevate the incidence of dengue.

Furthermore, whereas monthly data have been used in most previous time series studies, the weekly data used in the present VAR method provided more detailed associations between variables. Nonetheless, the present data indicate that meteorological variables do not affect dengue incidence. Presumably, meteorological data are insufficient to explain regional and other complex factors that influence dengue incidence.

A disadvantage of this study is the absence of data corresponding to the four viral serotypes DV-1, DV-2, DV-3, and DV-4, which may have differential influences on population immunity. In Sri Lanka, DV-2 and DV-3 are currently the most common serotypes. Further time series studies are required to decipher the combined effects of serotype and climate on dengue incidence. In this study, we used time series analysis and developed statistical approaches to determine the impact of meteorological variables on dengue incidence in Sri Lanka. Further time series studies may include other complex factors such as population density, forest cover rate, and socio–economic status. We were unable to add the data of population density or the immigration and emigration ratio to this time series analyses because migration data were not reported at weekly intervals. Although the usage of data related to demography has been attempted in the study by time series analysis, most of these studies gave up this use owing to difficulty in obtaining this type of demographic data in a short interval.

In Sri Lanka, census is conducted only once for approximately 10 years, and the population of other years is estimated. According to the last two censuses (2001 and 2012) by the Department of Census and Statistics in Sri Lanka, the average annual growth rate in Ratnapura, Colombo, and Anuradhapura from 2001 to 2012 is 0.59%, 0.35%, 1.33%, respectively. The highest annual growth rate in Sri Lanka between the 2001 to 2012 period was reported from Anuradhapura. In contrast, the annual growth rate in Ratnapura and Colombo is below 1%. Kalutara district (1.23%) and Gampaha district (1.02%) of the Western Province, including Colombo, have also reported annual population growth rates of more than 1%. It is appears that people migrate from the urban areas of Colombo to these two neighboring districts for residence, which explains the higher annual growth rates. Therefore, social demographic change in each area must be considered as the analyzing data in time series analysis. Meanwhile, we need to give a great deal of thought to the difficulty in collecting demographic data in the case of short-interval time series analysis such as that in this study.

## References

[1] Rajapakse S, Rodrigo C, Rajapakse A (2012) Treatment of dengue fever. Infect Drug Resist 5: 103–112. 22870039 10.2147/IDR.S22613 PMC3411372

[2] Rasgon JL (2011) Dengue fever: Mosquitoes attacked from within. Nature 476: 407–408. 21866151 10.1038/476407a

[3] Brady OJ, Gething PW, Bhatt S, Messina JP, Brownstein JS, et al (2012) Refining the global spatial limits of dengue virus transmission by evidence-based consensus. PLoS Negl Trop Dis 6: e1760. 22880140 10.1371/journal.pntd.0001760 PMC3413714

[4] WHO (2009) Dengue guidelines for diagnosis, treatment, prevention and control: World Health Organization. 1–147 p. 23762963

[5] Chastel C (2012) Eventual role of asymptomatic cases of dengue for the introduction and spread of dengue viruses in non-endemic regions. Front Physiol 3: 70. 22479252 10.3389/fphys.2012.00070 PMC3315825

[6] Weaver SC, Vasilakis N (2009) Molecular evolution of dengue viruses: contributions of phylogenetics to understanding the history and epidemiology of the preeminent arboviral disease. Infect Genet Evol 9: 523–540. 19460319 10.1016/j.meegid.2009.02.003 PMC3609037

[7] Mohammed HP, Ramos MM, Rivera A, Johansson M, Munoz-Jordan JL, et al (2010) Travel-associated dengue infections in the United States, 1996 to 2005. J Travel Med 17: 8–14. 20074096 10.1111/j.1708-8305.2009.00374.x

[8] WHO (2010) Communicable disease epidemiological profile, Sri Lanka.

[9] Epidemiology Unit MoH, Sri Lanka(2011) Surveillance case definitions for nortifiable diseases in Sri Lanka 2nd edition.

[10] Epidemiology Unit MoH, Sri Lanka (2011) Epidemiology bulletin Sri Lanka: Third Quarter 2011. 1–20p.

[11] Egbendewe-Mondzozo A, Musumba M, McCarl BA, Wu X (2011) Climate change and vector-borne diseases: an economic impact analysis of malaria in Africa. Int J Environ Res Public Health 8: 913–930. 21556186 10.3390/ijerph8030913 PMC3083677

[12] Huang F, Zhou S, Zhang S, Wang H, Tang L (2011) Temporal correlation analysis between malaria and meteorological factors in Motuo County, Tibet. Malar J 10: 54. 21375751 10.1186/1475-2875-10-54 PMC3060153

[13] Haque U, Hashizume M, Glass GE, Dewan AM, Overgaard HJ, et al (2010) The role of climate variability in the spread of malaria in Bangladeshi highlands. PLoS One 5: e14341. 21179555 10.1371/journal.pone.0014341 PMC3002939

[14] Constantin de Magny G, Thiaw W, Kumar V, Manga NM, Diop BM, et al (2012) Cholera outbreak in senegal in 2005: was climate a factor? PLoS One 7: e44577. 22952995 10.1371/journal.pone.0044577 PMC3432123

[15] Traerup SL, Ortiz RA, Markandya A (2011) The costs of climate change: a study of cholera in Tanzania. Int J Environ Res Public Health 8: 4386–4405. 22408580 10.3390/ijerph8124386 PMC3290983

[16] Constantin de Magny G, Murtugudde R, Sapiano MR, Nizam A, Brown CW, et al (2008) Environmental signatures associated with cholera epidemics. Proc Natl Acad Sci U S A 105: 17676–17681. 19001267 10.1073/pnas.0809654105 PMC2584748

[17] Ben-Ari T, Neerinckx S, Gage KL, Kreppel K, Laudisoit A, et al (2011) Plague and climate: scales matter. PLoS Pathog 7: e1002160. 21949648 10.1371/journal.ppat.1002160 PMC3174245

[18] Xu L, Liu Q, Stige LC, Ben Ari T, Fang X, et al (2011) Nonlinear effect of climate on plague during the third pandemic in China. Proc Natl Acad Sci U S A 108: 10214–10219. 21646523 10.1073/pnas.1019486108 PMC3121851

[19] Ari TB, Gershunov A, Tristan R, Cazelles B, Gage K, et al (2010) Interannual variability of human plague occurrence in the Western United States explained by tropical and North Pacific Ocean climate variability. Am J Trop Med Hyg 83: 624–632. 20810830 10.4269/ajtmh.2010.09-0775 PMC2929061

[20] de Magny GC, Thiaw W, Kumar V, Manga NM, Diop BM, et al (2012) Cholera outbreak in Senegal in 2005: was climate a factor? PLoS One 7: e44577. 22952995 10.1371/journal.pone.0044577 PMC3432123

[21] Honda Y, Ono M (2009) Issues in health risk assessment of current and future heat extremes. Glob Health Action 2.. 10.3402/gha.v2i0.2043 PMC2799309 20052374

[22] Reller ME, Bodinayake C, Nagahawatte A, Devasiri V, Kodikara-Arachichi W, et al (2012) Unsuspected dengue and acute febrile illness in rural and semi-urban southern Sri Lanka. Emerg Infect Dis 18: 256–263. 22304972 10.3201/eid1802.110962 PMC3310451

[23] Weerakoon KG, Kularatne SA, Edussuriya DH, Kodikara SK, Gunatilake LP, et al (2011) Histopathological diagnosis of myocarditis in a dengue outbreak in Sri Lanka, 2009. BMC Res Notes 4: 268. 21798066 10.1186/1756-0500-4-268 PMC3160397

[24] Tissera HA, Ooi EE, Gubler DJ, Tan Y, Logendra B, et al (2011) New dengue virus type 1 genotype in Colombo, Sri Lanka. Emerg Infect Dis 17: 2053–2055. 22099096 10.3201/eid1711.101893 PMC3310553

[25] Kanakaratne N, Wahala WM, Messer WB, Tissera HA, Shahani A, et al (2009) Severe dengue epidemics in Sri Lanka, 2003–2006. Emerg Infect Dis 15: 192–199. 19193262 10.3201/eid1502.080926 PMC2662655

[26] Kularatne SA, Pathirage MM, Kumarasiri PV, Gunasena S, Mahindawanse SI (2007) Cardiac complications of a dengue fever outbreak in Sri Lanka, 2005. Trans R Soc Trop Med Hyg 101: 804–808. 17428513 10.1016/j.trstmh.2007.02.021

[27] Kumar S, Managi S, Matsuda A (2012) Stock prices of clean energy firms, oil and carbon markets: A vector autoregressive analysis. Energy Economics 34: 215–226.

[28] Opgen-Rhein R, Strimmer K (2007) Learning causal networks from systems biology time course data: an effective model selection procedure for the vector autoregressive process. BMC Bioinformatics 8 Suppl 2S3. 10.1186/1471-2105-8-S2-S3 PMC1892072 17493252

[29] Akinboade OA, Braimoh LA (2010) International tourism and economic development in South Africa: a Granger causality test. International Journal of Tourism Research 12: 149–163.

[30] Pesaran H, Shin Y (1998) Generalized impulse response analysis in linear multivariate models. Economics Letters 58: 17–29.

[31] Ji X-j, Zhang Y-q, Hao L-y (2012) An Empirical Analysis of the Factors Affecting the Revenue of Shandong Province. Advances in Information Technology and Management 2: 268–272.

[32] Gharbi M, Quenel P, Gustave J, Cassadou S, La Ruche G, et al (2011) Time series analysis of dengue incidence in Guadeloupe, French West Indies: forecasting models using climate variables as predictors. BMC Infect Dis 11: 166. 21658238 10.1186/1471-2334-11-166 PMC3128053

[33] Descloux E, Mangeas M, Menkes CE, Lengaigne M, Leroy A, et al (2012) Climate-based models for understanding and forecasting dengue epidemics. PLoS Negl Trop Dis 6: e1470. 22348154 10.1371/journal.pntd.0001470 PMC3279338

[34] Pinto E, Coelho M, Oliver L, Massad E (2011) The influence of climate variables on dengue in Singapore. Int J Environ Health Res 21: 415–426. 21557124 10.1080/09603123.2011.572279

[35] Hii YL, Rocklov J, Ng N, Tang CS, Pang FY, et al (2009) Climate variability and increase in intensity and magnitude of dengue incidence in Singapore. Glob Health Action 2. 10.3402/gha.v2i0.2036 PMC2799326 20052380

